# The Unconventional Xer Recombination Machinery of *Streptococci/Lactococci*

**DOI:** 10.1371/journal.pgen.0030117

**Published:** 2007-07-13

**Authors:** Pascal Le Bourgeois, Marie Bugarel, Nathalie Campo, Marie-Line Daveran-Mingot, Jessica Labonté, Daniel Lanfranchi, Thomas Lautier, Carine Pagès, Paul Ritzenthaler

**Affiliations:** 1 Laboratoire de Microbiologie et Génétique Microbienne, CNRS, Université Paul Sabatier, Toulouse, France; 2 Department of Genetics, Biomolecular Sciences and Biotechnology Institute, University of Groningen, Haren, The Netherlands; National Cancer Institute, United States of America

## Abstract

Homologous recombination between circular sister chromosomes during DNA replication in bacteria can generate chromosome dimers that must be resolved into monomers prior to cell division. In *Escherichia coli,* dimer resolution is achieved by site-specific recombination, Xer recombination, involving two paralogous tyrosine recombinases, XerC and XerD, and a 28-bp recombination site *(dif)* located at the junction of the two replication arms. Xer recombination is tightly controlled by the septal protein FtsK. XerCD recombinases and FtsK are found on most sequenced eubacterial genomes, suggesting that the Xer recombination system as described in E. coli is highly conserved among prokaryotes. We show here that *Streptococci* and *Lactococci* carry an alternative Xer recombination machinery, organized in a single recombination module. This corresponds to an atypical 31-bp recombination site (*dif*_SL_) associated with a dedicated tyrosine recombinase (XerS). In contrast to the E. coli Xer system, only a single recombinase is required to recombine *dif*_SL_, suggesting a different mechanism in the recombination process. Despite this important difference, XerS can only perform efficient recombination when *dif*_SL_ sites are located on chromosome dimers. Moreover, the XerS/*dif*_SL_ recombination requires the streptococcal protein FtsK_SL_, probably without the need for direct protein-protein interaction, which we demonstrated to be located at the division septum of Lactococcus lactis. Acquisition of the XerS recombination module can be considered as a landmark of the separation of *Streptococci*/*Lactococci* from other firmicutes and support the view that Xer recombination is a conserved cellular function in bacteria, but that can be achieved by functional analogs.

## Author Summary

In bacteria, genetic information is mainly carried by a single circular chromosome. The replication of this circular molecule sometimes leads to the formation of a chromosome dimer unable to segregate in the daughter cells during the division process. In the bacterial model E. coli, chromosome dimers are resolved in monomers by site-specific recombination: two recombinases (XerC and XerD) cooperatively catalyze the recombination at a chromosomal site *(dif),* located at the junction of the two replication arms. This recombination is intimately coupled to cell division by the control of the septal protein FtsK. Xer recombination machinery as described in *E. coli* appears highly conserved among bacterial species. We show by comparative genomics and genetic studies that *Streptococci* use an alternative Xer recombination system, renamed XerS/*dif*_SL_, which is composed of a single recombinase phylogenetically unrelated to XerCD proteins and a noncanonical *dif* site. We also demonstrate that the streptococcal FtsK protein localizes at the division septum and operates the XerS/*dif*_SL_ recombination. This is the first identification of an alternative Xer recombination system in prokaryotes to out knowledge, which might indicate that other unusual chromosome dimer resolution systems could exist in bacterial phyla where a canonical *dif* site is not detected.

## Introduction

Chromosome replication is a key function in living cells, and any factor that impedes progression of replication forks can result in mutagenesis and genome instability. Several strategies have evolved to rescue replication forks stalled by DNA damage. Most of these depend on homologous recombination pathways but are not necessarily accompanied by strand exchange [1]. However, in cases where replication fork repair does lead to sister chromatid exchange, bacteria with circular chromosome(s) are faced with a potential topological problem because an odd number of crossovers between sister chromatids generates chromosome dimer, which must be converted back to monomers for a correct segregation to daughter cells. In *E. coli,* chromosome dimer formation occurs in 15% of the cell population [2,3], and conversion to monomers is carried out by the Xer site-specific recombination system (for recent reviews see [4,5]). This is composed of two paralogous tyrosine recombinases (integrases), XerC and XerD, which cooperatively catalyze strand exchanges at a 28-bp DNA sequence, the *dif* site, which must be located at the junction of the two replichores to be functional [3,6–8]. Xer recombination is intimately coupled to cell division [9] through the septal protein FtsK [10–12], a DNA translocase [8] with an essential N-terminal transmembrane domain involved in its localization at the septum [13], and a C-terminal DNA motor domain involved in positioning and synapsing the two *dif* sites of the chromosome dimer at the division septum [12,14–19] as well as in activating the strand exchange [8] by direct interaction with XerD [20,21].

Homologs of XerCD recombinases and FtsK are found in most eubacterial phyla and some archeal lineages [22] as well as the canonical *dif* site [23]. Moreover, interactions between the *E. coli dif* site and the XerCD recombinases of Haemophilus influenzae [24], Pseudomonas aeruginosa [25], Bacillus subtilis [26], Proteus mirabilis [27], and Caulobacter crescentus [28] have been experimentally demonstrated in vitro. These observations led to the general view that Xer recombination is a function conserved among bacteria harboring circular chromosome(s). However, regulation of strand exchange may differ, depending on the bacterial species: FtsK-mediated activation of Xer recombination in H. influenzae obeys the E. coli paradigm [21], whereas in *B. subtilis,* the model bacteria for firmicutes (formerly known as low GC-content Gram-positive bacteria), neither of the two FtsK analogs (SpoIIIE and YtpT) appears able to drive Xer recombination [26]. Several attempts have been made to identify the Xer recombination machinery in *Streptococci,* a taxonomic group belonging to firmicutes and comprising major pathogens [29] as well as innocuous food-grade species of major industrial importance [30,31]. These studies revealed putative XerCD recombinases but failed to identify a *dif* site [32,33].

We show here, by comparative genomics and functional analyses in *L. lactis, S. pneumoniae,* and *E. coli,* that *Streptococci* possess alternative Xer recombination machinery phylogenetically unrelated to the classical XerCD/*dif* system. This machinery involves a single tyrosine recombinase (XerS) and an atypical *dif* site *(dif*_SL_ *),* both located on a single genetic module. We also show that, in contrast to *B. subtilis,* the streptococcal FtsK protein localizes at the division septum and controls the XerS/*dif*_SL_ recombination.

## Results

### Identification of the Streptococcal *dif* Site by Comparative Genomics

**Figure 1:**
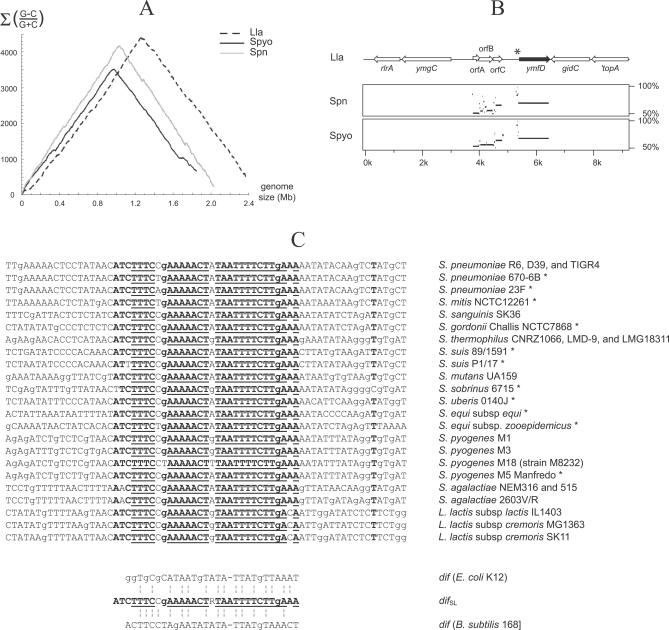
Identification of the Streptococcal *dif*_SL_ Site by Comparative Genomics (A) Cumulative GC skew diagrams of three streptococcal genomes. Lla, L. lactis IL1403; Spyo, S. pyogenes M1 GAS; Spn, S. pneumoniae R6. (B) Multiple DNA comparison is presented for the 10-kb regions encompassing the region of GC skew shift. The result is scaled to the gene organization of the L. lactis IL1403 *terC* region, and the conserved ORF is indicated in black. The lactococcal ORFs A, B, and C, not found in other streptococcal genomes, showed significant homology to the YmfD protein when fused together, suggesting ancient duplication of the *ymfD* region in L. lactis. The conserved ∼50-bp sequence is indicated by an asterisk. (C) Sequence similarity of putative *dif*_SL_ sites from 49 publicly available streptococcal genomes (December 2006) and comparison with the E. coli and *B. subtilis dif* sites are presented. Nucleotides conserved in all except one species are indicated in bold, and those conserved in all species are indicated in bold and underlined. S. pyogenes M1 strains were M1 GAS, MGAS10750, MGAS5005, and MGAS10394. S. pyogenes M3 strains were MGAS315, MGAS2096, MGAS10270, MGAS9429, MGAS6180, M49–591, and SSI-1. S. agalactiae sequences of strains A909, H36B, COH1, CJB111, and 18RS21 were identical to strain 2603V/R. Asterisks indicate unfinished genomes.

Assuming that Xer recombination is highly conserved in eubacteria with a significant homology of the *dif* sites even between distantly related species [26], we performed an in silico search for putative *dif* sites in several completely sequenced firmicutes genomes. Candidate *dif* sites should: (i) have a significant similarity with that of *B. subtilis (dif*_BS_ *),* (ii) occur only once per genome, and (iii) be localized in the replication terminus *(terC),* defined as the chromosomal region located opposite the replication origin *(oriC)* where compositional strand biases switch sign [34,35]. Using these rules, a canonical *dif* site could be identified in all species analyzed except for *Streptococci* and *Lactococci* (Table 1). We therefore used an alternative three-step approach based on comparative genomics to identify the streptococcal/lactococcal *dif* site (Figure 1). The *terC* region for three streptococcal genomes was localized (Figure 1A) using a cumulative GC skew diagram [34], and a comparison of the 10-kb region encompassing the GC skew shift was performed (Figure 1B). This analysis revealed a 2-kb segment that showed significant similarity within the three species (>70% identity at the DNA level) and included a 356-amino-acid tyrosine-recombinase–encoding gene (annotated *ymfD* on the L. lactis IL1403 genome [36] but hereafter renamed *xerS*) preceded by a ∼50-bp highly (>90%) conserved sequence. When used to scan 49 genomes of other streptococcal species (Figure 1C), this ∼50-bp fragment revealed a 31-bp consensus sequence (hereafter named *dif*_SL_) with weak homology to the B. subtilis or *E. coli dif* sites, but with an overall structure resembling the DNA targets for tyrosine recombinases (i.e., two imperfect inverted repeats separated by a 6–8-bp central sequence).

**Table 1:**
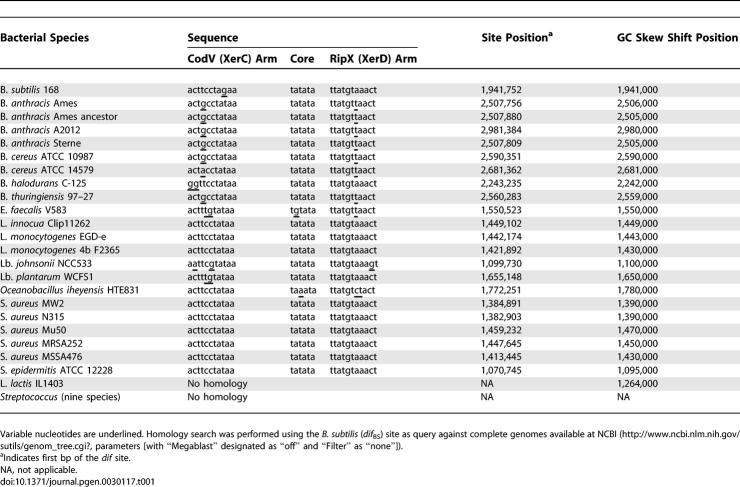
Localization of *dif* Sites in Some Firmicutes

**Figure 2:**
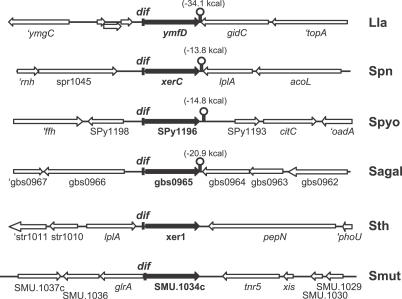
Gene Context Analysis of the 10-kb *terC* Region of Different Streptococcal Species Lla, L. lactis IL1403; Spn, S. pneumoniae R6; Spyo, S. pyogenes M1 GAS; Sagal, S. agalactiae NEM316; Sth, S. salivarius subsp*. thermophilus* CNRZ1066; Smut, S. mutans UA159. The ORF coding for the putative tyrosine recombinase is shown in black. Only putative ρ-independent transcription terminators with free energy (ΔG) <−12 kcal.mol^−1^ are indicated.

Comparative analysis of the genetic context in the 10-kb *terC* region of different streptococcal species revealed notable features strongly suggesting that streptococcal Xer recombination machinery is defined by one genetic module, corresponding to the *dif*_SL_ site followed by one of its dedicated recombinases (Figure 2). The physical link between *dif*_SL_ and *xerS* open reading frame (ORF) was found to be preserved among all *Streptococci* for which there is sequence data, and no genetic element other than *dif*_SL_-*xerS* was conserved in the 10-kb *terC* region. In addition, the genes surrounding *dif*_SL_-*xerS* did not show a preferred orientation that might indicate possible cotranscription with the *xerS* gene. Moreover, the *xerS* ORF often exhibits a putative ρ-independent transcription terminator at its end. These observations indicate that *xerS* is unlikely to be part of an operon and suggest that the *dif*_SL_-*xerS* pair behaves as an individual genetic module.

### In Vivo Characterization of the *dif*_SL_ Site

The candidate *dif*_SL_ site was tested for its ability to support site-specific recombination in L. lactis and S. pneumoniae by intermolecular recombination assays. A 37-bp synthetic sequence encompassing the putative 31-bp lactococcal *dif*_SL_ site was cloned in a plasmid that does not replicate in firmicutes (pCL52, Table S1). The resulting construction (pCL235, Table S1) was used to transform a wild-type (WT) strain of L. lactis (MG1363, Table S1). In contrast to pCL52, which did not yield transformant, pCL235 produced transformants at an efficiency representing 1% of the efficiency attained with a replicative plasmid (unpublished data). This demonstrates that the putative *dif*_SL_ site was capable of rescuing pCL235, presumably by promoting integration of exogenous DNA into the lactococcal chromosome. When transformed into a *recA* strain (VEL1122, Table S1), plasmid pCL235 was also rescued with the same efficiency as in the WT strain (unpublished data), indicating that plasmid integration occurred in a RecA-independent manner. Moreover, as judged by Pulsed-Field gel Electrophoresis analysis (Figure S1, lanes 2 and 4), pCL235 integrated into the chromosome of both strains at the location predicted for the native *dif*_SL_ site. Thus, the 37-bp sequence appeared to contain the DNA target of a site-specific recombination system. The *dif*_SL_-mediated site-specific integration was also demonstrated to be a general process in *Streptococci*, since plasmid pGh9, a temperature sensitive replication *(repA* ^ts^ *)* mutant containing either the lactococcal 37-bp sequence described above (pCL231, Table S1) or its pneumococcal counterpart (pCL403, Table S1), integrated into the chromosomes of L. lactis and S. pneumoniae under nonpermissive conditions with comparable efficiencies (respectively 4.88 × 10^−2^ [± 2.33 × 10^−2^] cell^−1^ and 2.67 × 10^−2^ [± 1.55 × 10^−2^] cell^−1^). However, it should be mentioned that location of the insertion site of pCL403 has not been verified in S. pneumoniae.

The minimal size of the *dif*_SL_ site was determined in L. lactis by scoring the integration efficiency of pGh9 containing variants of the *dif*_SL_ site (Table 2). Reducing the length of the *dif*_SL_ region from 48 to 31 bp did not alter the plasmid integration efficiency, indicating that the strongly conserved T located 13 bp away from the 31-bp consensus sequence (Figure 1C) was not part of the *dif*_SL_ site. However, removing the two external bp from both sides of the 31-bp consensus sequence (Table 2, *dif*-8) led to a 100-fold decrease in integration efficiency, though this sequence was still proficient in site-specific recombination at the native *dif*_SL_ site (Figure S1, lanes 3 and 5). Finally, deleting two nucleotides at either side of the 31-bp consensus sequence led to a 2-fold (Table 2, compare *dif*-7 to *dif*-5; Wilcoxon test, *p* < 0.003) or 4-fold (Table 2, compare *dif*-6 to *dif*-5; Wilcoxon test, *p* < 0.01) reduction in integration frequency. Together, these results led us to propose that the 31-bp consensus sequence defines the authentic *dif*_SL_ site.

**Table 2:**
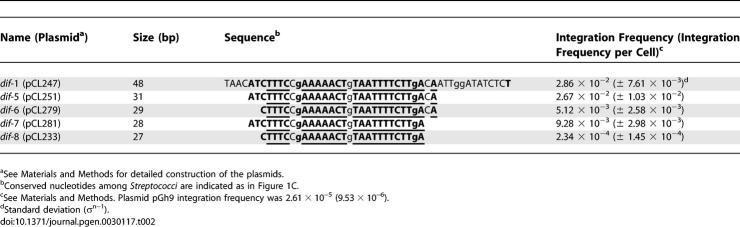
Chromosomal Integration Frequencies of *dif*_SL_ Variants in L. lactis

### Recombination at *dif*_SL_ Requires One Recombinase, XerS

**Figure 3:**
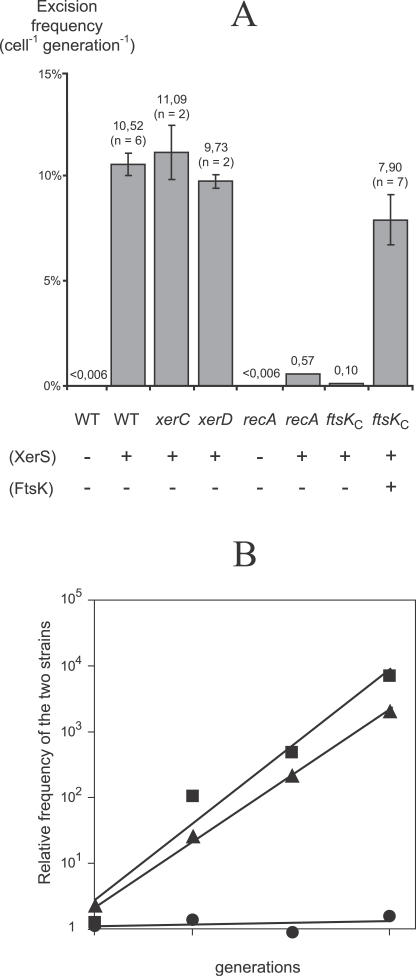
XerS/*dif*_SL_ Recombination in E. coli (A) Km^R^ cassette excision mediated by XerS in E. coli in different genetic backgrounds is shown. The +/− signs indicated presence/absence of plasmid expressing the *L. lactis xerS* (pCL297) or the *E. coli ftsK* (pCL263) genes. When available the number of independent experiments *(n)* is indicated below each excision frequency mean value. Error bars correspond to the standard deviation (σ^n−1^). (B) Effect of the lactococcal XerS/*dif*_SL_ system on chromosome dimer resolution in E. coli as measured by growth competition assays is presented. E368 (XerS ^+^ *dif*_SL_^+^) was mixed with E367 (XerS ^+^ *dif*_SL_^−^, squares), E378 (XerS^+^ *dif*_SL_^+^ *ftsK*_C_^−^, triangles), or E375 (WT strain with XerS, circles) at a ratio of 1:1 and grown in serial culture for 60 generations. Values were calculated from two independent assays.

Given that predictive analyses revealed XerS as the prime candidate for the actual Xer recombinase, a recombination assay was performed in S. pneumoniae to test if XerS was needed for recombination at *dif*_SL_. This streptococcal species was selected mainly for its facility to construct mutants compared to L. lactis. The recombination at the *dif*_SL_ site was totally abolished in a *xerS* mutant (strain S501, Table S1; Materials and Methods), with undetectable integration of pCL403, demonstrating that XerS was one catalytic recombinase of the XerS/*dif*_SL_ system. To test whether XerS was the only recombinase involved in the Xer catalytic machinery, XerS/*dif*_SL_ recombination was assayed in E. coli using an excision assay previously developed for the genetic analysis of the *E. coli dif* site activity [37]. Briefly, the native *E. coli dif* site was replaced by a cassette containing two directly repeated lactococcal *dif*_SL_ sites flanking a kanamycin resistance (Km^R^) gene (strain E359, Table S1), and the excision frequency (cell^−1^ generation^−1^; Materials and Methods) was determined by counting the number of Km^S^ recombinants at different generations during serial cultures (Figure 3A). In absence of the lactococcal XerS recombinase, almost no recombination was observed (<0.006% cell^−1^ generation^−1^) indicating that XerCD of E. coli do not recombine *dif*_SL_. In contrast, introduction of a plasmid expressing the lactococcal *xerS* gene (pCL297, Table S1) increased the excision frequency to 10% cell^−1^ generation^−1^ (Figure 3A), a value close to the excision frequency observed in *E. coli* when using the native XerCD/*dif* system [3]. In addition, the excision frequency was not significantly altered in *E. coli xerC* or *xerD* mutant (Figure 3A). This indicates that fortuitous interaction between XerS and E. coli XerC or XerD recombinases is unlikely to account for recombination at *dif*_SL_ sites. However, as recombination assay has not been performed on a *xerC xerD* double mutant, this cannot be totally ruled out. XerS also promoted intermolecular recombination between one lactococcal *dif*_SL_ site replacing the native *E. coli dif* site on the chromosome (strain E368, Table S1) and a second *dif*_SL_ site located on a nonreplicative plasmid (unpublished data). Together, these data demonstrated that XerS is the only streptococcal tyrosine recombinase required to catalyze intra- and intermolecular recombination between *dif*_SL_ sites.

A phylogenetic analysis of all tyrosine recombinases present in the sequenced genome of five streptococcal species revealed another integrase conserved among *Streptococci*. This atypical recombinase, more related to phages' integrases (Figure S2) and previously identified as XerD in S. pneumoniae [33], lacks the extreme C-terminal region and two amino acids of the catalytic tetrad R-H-R-Y [38]. When tested alone in E. coli, YnbA (the lactococcal ortholog of S. pneumoniae XerD) showed no intra- or intermolecular recombination activity on *dif*_SL_ and did not influence the recombination process when coexpressed with XerS. Moreover, it did not bind specifically to the lactococcal *dif*_SL_ site in vitro (unpublished data). Therefore, YnbA is unlikely to belong to the streptococcal Xer system.

### XerS/*dif*_SL_ and Chromosome Dimer Resolution in E. coli

Although the XerS/*dif*_SL_ system involves only one recombinase, as do the Cre/*loxP* and Flp/FRT systems, its location at the *terC* region of streptococcal chromosomes strongly suggests that it functions to resolve chromosome dimers. To examine whether XerS/*dif*_SL_ can substitute the XerCD/*dif* system in *E. coli,* we used the growth competition assays (Figure 3B) previously developed to show that XerCD/*dif* resolved chromosome dimers in E. coli [3,39]. For that purpose, we constructed E. coli strains containing or not one lactococcal *dif*_SL_ site replacing the native *dif* site. The strain harboring a complete streptococcal Xer system (E368, Table S1) showed a growth advantage of 10% generation^−1^ when competed with its isogenic strain missing the *dif*_SL_ site (E367, Table S1). As found for XerCD in E. coli [37], this selective benefit matches the excision frequency of the Km^R^ cassette in the excision assay described above. In addition, strain E368 showed no growth defect compared to a strain harboring a functional XerCD/*dif* system (E375, Table S1). These results were correlated with cell morphology changes: strain E367 retained the filamentation phenotype of an E. coli strain defective in Xer recombination, while the strain harboring the complete XerS/*dif*_SL_ system displayed a WT cell morphology (unpublished data). Thus, the XerS/*dif*_SL_ system can substitute XerCD/*dif* in E. coli to resolve chromosome dimers.

Chromosome dimers in E. coli are mostly formed by homologous recombination [2]. As a *recA* mutation also drastically reduces the XerCD-mediated recombination at *dif* [2,37], this argued toward the fact that chromosome dimer is mandatory for creating the conditions necessary for a recombination between two directly repeated *dif* sites. Such dependence was investigated for the XerS system using the same E. coli excision assay [37]. The frequency of the Km^R^ cassette excision by the XerS system fell from 10% to less than 0.6% cell^−1^ generation^−1^ (Figure 3A) in a *recA* derivative of the E359 strain (strain E379, Table S1). As this 20-fold reduction of recombination efficiency was similar to that observed in E. coli [37], this strongly suggests that, as for the XerCD/*dif* system, XerS can perform efficient recombination only when *dif*_SL_ sites are located on chromosome dimers.

### XerS/*dif*_SL_ Recombination Depends on the Septal Protein FtsK

**Figure 4:**
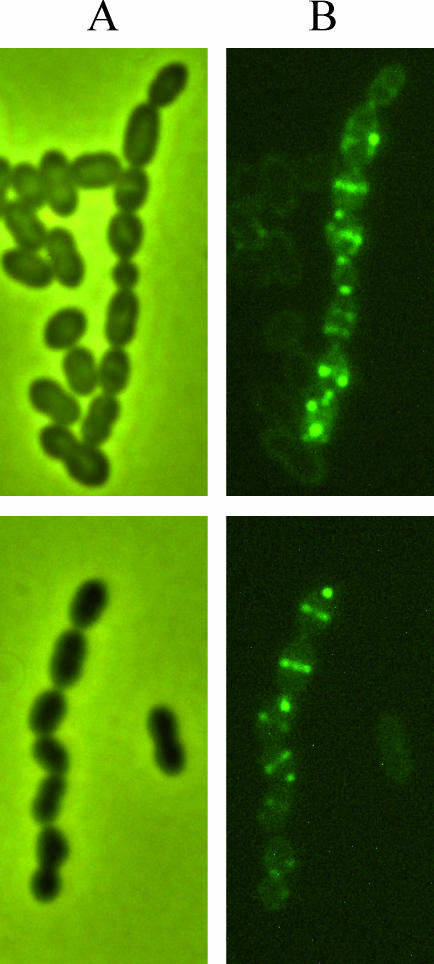
Subcellular Localization of FtsK_SL_-GFP Proteins in L. lactis Phase-contrast (A) and fluorescence (B) microscopy of L. lactis NZ900 overexpressing full-length lactococcal FtsK-GFP (upper) or N-ter Ftsk-GFP (lower) are presented. Cells were analyzed by microscopy on midexponential growth phase.

All streptococcal genomes sequenced so far contain one ORF encoding a protein homologous to the 787-amino-acid B. subtilis protein SpoIIIE. These SpoIIIE-like proteins (hereafter named FtsK_SL_) range from 758 *(S. mutans)* to 816 *(S. agalactiae)* amino acids in length and retain the structural signatures of proteins from the FtsK-HerA superfamily [40]: they contain a ∼180-amino-acid N-terminal region of weak similarity that includes four predicted transmembrane domains and a strongly conserved ∼500-amino-acid C-terminal region corresponding to the DNA translocase domain (unpublished data). The cellular localization of FtsK_SL_ was determined in L. lactis using GFP fusions, corresponding to the GFP protein fused to the C-terminal of the full-length (FtsK_1 − 763_ − GFP) or N-terminal region (FtsK_1 − 181_ − GFP) of lactococcal FtsK_SL_. Both GFP fusions clearly localized at the septum of L. lactis (Figure 4), indicating that as expected, FtsK_SL_ localizes at the lactococcal division septum and its 181 amino acids containing the four transmembrane domains were sufficient to drive this localization.

The control of XerS-mediated recombination by FtsK was examined in S. pneumoniae and E. coli. For that purpose, strains expressing FtsK proteins deleted of their C-terminal part were constructed (*ftsK*_C_ mutants, only the first 405 amino acids and the first 316 amino acids of FtsK are synthesized in S. pneumoniae and E. coli respectively; Materials and Methods). Surprisingly, during the construction of the *ftsKC* mutants in *S. pneumoniae,* insertions of the Mariner minitransposon were obtained into the C-terminal or the N-terminal domain of FtsK_SL_. This suggests that neither the C- nor the N terminus is essential for the growth of this bacterium, though all pneumococcal *ftsK* mutants were severely impaired in growth rate and cell viability as *xerS* mutants (unpublished data). In *S. pneumoniae,* XerS/*dif*_SL_ recombination depended on the C-terminal part of FtsK_SL_, because integration of the *repA* ^ts^ plasmid containing the pneumococcal *dif*_SL_ site (pCL403), though not totally abolished as for the *xerS* mutant, became severely impaired in the *ftsK*_C_ mutant (S502, Table S1), with an efficiency of 1.52 × 10^−4^ (± 3.5 × 10^−4^) cell^−1^ corresponding to less than 1% of the integration efficiency of the WT strain. Similar observations were made in the *ftsK*_C_ mutant of E. coli (E372, Table S1), using the excision assay. The excision frequency of the *dif*_SL_-Km^R^- *dif*_SL_ cassette dropped from 10% in WT strain to 0.1% cell^−1^ generation^−1^ in the *ftsK*_C_ strain (Figure 3A). This decrease was unambiguously associated to the lack of the C-terminal part of FtsK, since expression of the full-length *E. coli ftsK* gene carried on a pBAD-derivative plasmid (pCL263, Table S1) restored Km^R^ cassette excision to nearly the WT frequency (Figure 3A). In addition, results from growth competition assays (Figure 3B) or cell morphology observations (unpublished data) also showed that the XerS system was unable to resolve chromosome dimers in E. coli in absence of the C-terminal domain of FtsK. Together our data demonstrated that XerS/*dif*_SL_ recombination in S. pneumoniae and *E. coli,* as well as dimer resolution in *E. coli,* depends on FtsK.

## Discussion

In this work, we provide experimental evidence that *Streptococci* possess an unconventional Xer recombination machinery that requires only one tyrosine recombinase, XerS, to catalyze the site-specific recombination at a 31-bp sequence *dif*_SL_. This raises an important question as to whether this system is orthologous to the “classical” E. coli XerCD system found in most bacterial species, including many other firmicutes. Not only does XerS differ significantly in length and primary sequence from members of the XerCD recombinases family (unpublished data), but *dif*_SL_ also differs in length and shows a weak similarity with the E. coli or *B. subtilis dif* sites (Figure 1C). Moreover, *dif*_SL_ is located immediately upstream the *xerS* coding sequence in all streptococcal and lactococcal species analyzed. Such genetic organization contrasts with that of classical XerCD systems, with the two recombinases encoded by genes located far from each other and distant from *dif,* and is more comparable to the integration modules of mobile elements such as integrons [41], bacteriophages such as P1 [42] or mycobacteriophage L5 [43], and some ICEs such as the *clc* element from *Pseudomonas* [44] or CTnDOT from *Bacteroides* [45]. As *Streptococci* and *Lactococci* (together defining the taxonomic family of *Streptococcaceae*) represent a rather homogeneous phylogenetic group among firmicutes when compared to other genera such as *Clostridium* or *Lactobacillus* [46,47], we speculate that acquisition of the XerS system might have replaced the “classical” XerCD system at the time of or soon after their split from other firmicutes, with this event representing one landmark of this separation.

As demonstrated in this study, the *cis*-organization of the *dif*_SL_-*xerS* module is not mandatory for efficient recombination, but this probably reveals a selective pressure to maintain that arrangement. Although at present the *xerS* transcription start point location is unknown, we speculate that *dif*_SL_ either lies between the *xerS* ORF and its promoter or is part of the *xerS* promoter. If this is true, this unusual arrangement might reflect a regulatory mechanism in which binding of XerS to *dif*_SL_ might autoregulate *xerS* expression. Alternatively, as it has been recently observed that some filamentous phages [48] or genetic islands [49] can divert the XerCD recombination system to integrate themselves at the chromosomal *dif* site of several bacteria, another hypothesis could be that the *dif*_SL_-*xerS* arrangement might serve to prevent insertion of additional genetic material at *dif*_SL_, because such event would separate the *xerS* ORF from its promoter and lead to inactivation of the chromosome dimer resolution system.

With only one catalytic recombinase involved in the recombination reaction, the XerS system is more similar to Cre/*loxP* and Flp/FRT than to XerCD/*dif*. However, XerS retains particular features that could indicate alternative mechanism in the recombination process. For instance, in vivo characterization of the *dif*_SL_ site in L. lactis revealed an asymmetry in its organization, with left and right arms differing in length (the left arm being 2-bp longer than the right one) as well as in nucleotide sequence (Table 2). This differs from *loxP* and FRT sites, which contain two perfectly symmetrical 13-bp arms surrounding the core region [50,51]. How a single recombinase can accommodate dissimilar binding sites to perform the DNA strand exchange reaction without accessory factor has to be analyzed, but we speculate that asymmetry of the *dif*_SL_ site might have a role in the control of this strand exchange order.

Though we did not provide direct experimental data demonstrating that XerS/*dif*_SL_ is involved in chromosome dimer resolution in *Streptococci,* several lines of evidence strongly suggest that dimer resolution is the primary task of this site-specific recombination system. First, classical XerCD recombinases and canonical *dif* site are not present in streptococcal/lactococcal genomes but substituted by the XerS/*dif*_SL_ recombination module at the chromosomal location predicted for a site-specific recombination system acting on chromosome dimer resolution. Second, to catalyze the strand exchange reaction XerS seems to require at least one of the two *dif*_SL_ sites located on the chromosome, because recombination between two *dif*_SL_ sites contained within a multicopy plasmid with theta replication (pSC101 derivative in *E. coli,* and pAMB1 in *L. lactis,* unpublished data) could be detected neither in E. coli nor in L. lactis. At last, not only is XerS/*dif*_SL_ able to resolve chromosome dimers in E. coli as efficiently as the native XerCD/*dif* system (Figure 3B), but XerS/*dif*_SL_ recombination also hinged on formation of chromosome dimers, as revealed by the RecA-dependency of the Km^R^ cassette excision (Figure 3A), with the excision efficiency exactly matching the frequency of chromosome dimers resolution.

We also demonstrated that, in contrast to SpoIIIE from B. subtilis that only infrequently (∼6%) concentrates at the vegetative septum [52] and is not involved in the Xer recombination [26], the streptococcal FtsK_SL_ protein localizes at the division septum and still directs the XerS/*dif*_SL_ recombination, as dimer resolution and intra- or intermolecular recombination were drastically inhibited in E. coli and S. pneumoniae cells lacking the C-terminal part of FtsK. Although our preliminary analyses of the pneumococcal *ftsK* mutants need to be confirmed, the ability to obtain viable cells depleted of FtsK suggests that neither the N- nor the C terminus of the protein is essential in *Streptococci*. As essentiality of FtsK seems to be species dependent, with only the N-terminal part in E. coli and the C terminus in C. crescentus being essential [53,54], we hypothesize that activity of FtsK, though still involved in cell division and DNA translocation, could slightly differ among the different bacteria. However, it appears that no correlation can be done between essentiality and localization of FtsK to the division septum. In E. coli and some other γ-proteobacteria, the C-terminal part of FtsK drives the XerCD recombination reaction in two ways: by participating to the formation of the recombination synapse through its DNA translocase activity [14–16] and activating the recombination reaction by direct interaction with XerD [8]. Some of our data strongly indicated that such interaction between FtsK_SL_ and XerS is unnecessary to activate the XerS/*dif*_SL_ recombination in *Streptococci,* though this cannot be totally ruled out. First, the XerS-mediated intramolecular recombination frequency at *dif*_SL_ in E. coli (Figure 3A) was close to that measured with XerCD/*dif* [3], suggesting no species specificity for FtsK requirement. This observation contrasts to that made in E. coli where the H. influenzae FtsK was inefficient in activation of the E. coli XerD and vice versa, implying that the FtsK-XerD interaction is highly species specific in these bacteria [21]. In addition, both pneumococcal and lactococcal XerS protein sequences do not contain the amino acids motif (RQ–QQ) found in E. coli XerD and involved in its specific interaction between with FtsK [20]. At last, the cassette excision by recombination at *dif*_SL_ in *E. coli,* as well as plasmid integration in *S. pneumoniae,* was not totally abolished in *ftsK*_C_ mutants but dropped to 1% of the recombination activity of WT strains, suggesting that some productive recombination synapses might form independently of FtsK, most probably by the random collision of two *dif* sites. This observation also contrasts the results obtained with the cassette excision assay performed with the E. coli XerCD/*dif* system, wherein no recombination was detected in an *ftsK*_C_ mutant [55], suggestive of the FtsK-mediated activation of the recombination. Our data are more easily accommodated to a model where XerS is unable to form a productive synapse and requires the DNA translocase activity of FtsK_SL_ to bring the two *dif*_SL_ sites of a chromosome dimer close to each other and in an active geometry before performing the strand exchange. However, the recombination would not need direct activation by protein interaction between FtsK_SL_ and XerS. However, as for the XerCD model [11], our model cannot provide satisfactory explanation to how FtsK is involved in the intermolecular recombination between one *dif*_SL_ site located on a suicide (or *repA* ^ts^) plasmid and the chromosomal *dif*_SL_ site, and the mechanism of the FtsK_SL_-mediated control has to be analyzed further.

In conclusion, the discovery of a Xer recombination system phylogenetically unrelated to the classical XerCD system reinforces the idea that chromosome dimer resolution can be viewed as a housekeeping function conserved among bacteria with circular chromosome(s), but that some species can use functional analogs to perform this task. We expect that other bacterial species among those whose genome(s) are missing a canonical *dif* site also contain alternative chromosome dimer resolution systems. Finally, we note that the particularity of the XerS system makes it a valuable candidate for the development of new antibacterial drugs specifically directed against the pathogenic *Streptococci*.

## Materials and Methods

### Plasmids, bacterial strains, and growth conditions.

The plasmids and bacterial strains used in this study are listed in Table S1. E. coli strains and plasmids containing synthetic lactococcal or pneumococcal variants of *dif*_SL_ sites were constructed using the procedure provided in Text S1. E. coli strains were grown at 30 °C in LB medium. Antibiotics were used at the following concentrations: erythromycin (Em) 150 μg ml^−1^, chloramphenicol (Cm) 20 μg ml^−1^, spectinomycin (Spc) 100 μg ml^−1^, kanamycin (Km) 50 μg ml^−1^, and ampicillin (Ap) 25 μg ml^−1^. L. lactis strains were grown semi-anaerobically at 30 °C in M17 broth (Merck KGaA, http://www.merck.de) supplemented with 0.5% (w/v) glucose (GM17) and transformed as previously described [56]. Antibiotics used for selection of lactococcal transformants were: Em 1 μg ml^−1^, Cm 5 μg ml^−1^, and Spc 200 μg ml^−1^. S. pneumoniae strains were grown in Todd-Hewitt broth (Difco/BD Biosciences, http://www.bdbiosciences.com) supplemented with 0.5% yeast extract (THY) and transformed using synthetic competence-stimulating peptide (CSP) as described [57]. Antibiotic concentrations used for selection of pneumococcal transformants were: Em 0.2 μg ml^−1^ and Km 500 μg ml^−1^.

### DNA manipulation.

Restriction and modification enzymes were purchased from New England Biolabs (http://www.neb.com) and used as recommended by the supplier. Plasmid DNA from E. coli was isolated with the Qiaprep spin kit according to the manufacturer's instructions (Qiagen, http://www.qiagen.com). Chromosomal DNA from *E. coli, L. lactis,* and S. pneumoniae was isolated with the DNeasy tissue kit according to the manufacturer's instructions (Qiagen). Preparation of lactococcal genomic DNA embedded in agarose matrix, Pulsed-Field gel Electrophoresis, and Southern blot with dried agarose gels were performed as previously described [58]. Hybridization signals were detected with a Bioimaging BAS1000 analyzer system (FUJI Photo Film, http://www.fujifilm.com) and analyzed with TINA version 2.07c software (Raytest Isotopenβgeräte GmBH, http://www.raytest.de).

### Genome sequences analyses.

Nucleotide sequences were obtained from NCBI (http://www.ncbi.nlm.nih.gov/genomes/static/eub_g.html), JGI (http://genome.jgi-psf.org/tre_home.html), and the Sanger Institute (http://www.sanger.ac.uk/Projects/Microbes). Cumulative GC skews were obtained from an in-house build program (Laurent Lestrade, Laboratoire de Biologie Moléculaire des Eucaryotes, Toulouse, France). Multiple DNA comparison was performed using MultiPipMaker program [59].

### Intermolecular recombination assays in L. lactis and S. pneumoniae.

Chromosomal integration assays of *repA* ^ts^ plasmid pGh9 or *dif*_SL_-containing derivatives in L. lactis were performed according to [60]. For *S. pneumoniae,* frozen strains containing the pGh9 or derivatives were grown at 39 °C (water bath) to an OD_600_ = 0.3 in THY without antibiotics. Appropriate dilutions were plated on 10 ml of D medium [57] containing 2% of defibrinated horse blood (bioMérieux, http://www.biomerieux.com) and supplemented when appropriate with Em, and plates were incubated at 39 °C. Integration of the pGh9 plasmid was undetectable in S. pneumoniae (no colonies were observed when plating 0.1 ml of the undiluted bacterial culture). The integration frequency per cell (ipc) was calculated as the geometric mean of the ratio of colonies on selective versus nonselective plates obtained from five to 19 independent experiments.

### In vitro mariner mutagenesis in S. pneumoniae.

Mutagenesis was carried out as described [61]. The target DNA for in vitro transposition of the Km^R^ mariner minitransposon pR410 [61] were obtained by PCR reactions using R800 chromosomal DNA as template. The sizes of PCR fragments were: 2.012 bp for *xerS* gene (primers: forward, 5′-TAgAAAACCgATTCTCAgAAATgAgATC-3′; reverse, 5′-gAAgAAgAATTggCCgA AATCAA-3′) and 4.053 bp for *ftsK*_SL_ gene (primers: forward, 5′-AAAACAAAgCCTTggTggTgC CT-3′; reverse, 5′-CTTgCgACAAgAAAgggAAA-TTT-3′). The mutagenized PCR fragments were then transformed into strain R800. For each mutagenesis, ten Km^R^ transformants were checked by PCR and shown to carry a *mariner* insertion. The accurate insertion position of the transposon, as well as its orientation, was determined by PCR and DNA sequencing as described [61]. The resulting chromosome structures of the selected mutants were: R800 *xerS*, insertion of *mariner* 167 bp downstream the ATG (insertion allows the synthesis of only the first 55 amino acids of XerS); R800 *ftsK*_C_, insertion of *mariner* 1217 bp downstream the ATG (insertion allows the synthesis of only the first 405 amino acids of FtsK).

### *dif*_SL_-Km^R^-*dif*_SL_ cassette excision in E. coli.

XerS-mediated excision of the *dif*_SL_-Km^R^-*dif*_SL_ was performed according to [37]. Briefly, E. coli strains transformed with the XerS expression plasmid pCL297 (or cotransformed with pCL297 and pCL263) were growth in serial cultures at 30 °C (because of the thermosensitive replication of plasmid pCL297) for 60 generations in nonselective LB medium. The ratio of Km^R^/total colonies was measured each 20 generations, and excision frequencies (cell^−1^ generation^−1^) deduced according to Perals et al. [37]. For low excision frequencies (<1% cell^−1^ generation^−1^), such as those obtained in the absence of XerS in *recA* or *ftsK*_C_ single mutants and *recA ftsK*_C_ double mutants, 200 colonies picked from nonselective plates after 85 generations of serial growth were replica plated on Km-containing LB plates, the ratio of Km^R^/total colonies was measured, and used to estimate the excision frequencies per generation.

### Growth competition assays in E. coli.

Coculture experiments were performed as described by Perals et al. [3], except that the growth temperature was 30 °C (because of the thermosensitive replication of plasmid pCL297). The growth advantage per generation, corresponding to the frequency of chromosome dimer resolution, was calculated from the slope of each competition curve as according to [3]. The strains containing one *dif*_SL_ site located at the native position of the *E. coli dif* site correspond to Km^S^ strains obtained from replica plating from nonselective plates in the *dif*_SL_-Km^R^-*dif*_SL_ excision experiments described above.

### Subcellular localization of FtsK-GFP fusions in L. lactis.

The lactococcal *ftsK* gene was amplified by PCR from chromosomal DNA of L. lactis MG1363. The 557-bp PCR fragment (encoding the first 181 amino acids of FtsK; primers: forward, 5′-CATg**CCATgg**gTgAAAgTAAAAAAATgCCT-3′; reverse 5′-CCATCgATTTTAgggAAAAgTCC TggAA-3′) and the 2303-bp PCR fragment (full-length *ftsK* gene; primers: forward, 5′-CATg**CCATgg**gTgAAAgTAAAAAAATgCCT-3′; reverse 5′-CCATCgATCTCTTCTACTCCTC CAATA-3′) were cleaved with *Nco*I (bold) and *Eco*RV (underlined) and cloned into corresponding sites of pNG8048e [62], generating respectively pFtsKNter and pFtsKFL. The *Cla*I-*Xba*I fragment containing the *gfpmut1* gene of pSG1154 [63] was cloned into the corresponding sites of pFtsKNter and pFtsKFL yielding plasmids pKNtergfp and pKFLgfp respectively. For fluorescence microscopy, cells from an overnight culture were diluted (1:100) into fresh medium and grown at 30 °C with agitation. At OD_600_ = 0.5, supernatant of the nisin-producing strain NZ9700 [64] was added at dilution 1:1,000. At OD_600_ = 1, 2 μl of culture was directly applied to a polysine microscope slide (Omnilabo, http://www1.omnilabo.nl) and covered with a cover glass. Cells were examined using a 100× oil immersion objective on a Zeiss microscope (Carl Zeiss, http://www.zeiss.com), and the fluorescent signal of GFP was detected using filter set 09 (excitation, 450–490 nm and emission, > 520 nm) from Zeiss. Images were captured with an Axion Vision camera (Axion Technologies, http://www.axiontech.com), and assembly of the final figures used Adobe Photoshop version 6.0.

## Supporting Information

Figure S1 Physical Analysis of *dif*_SL_-Mediated Integration Site in L. lactis Chromosome Shown is Pulsed-Field gel Electrophoresis of *Sma*I-digested chromosomes (A) and corresponding Southern hybridization (B) of WT strain (MG1363) and its *recA*-derivative (VEL1122) after *dif*-mediated integration of plasmids pCL235 or pCL237. Lanes: 1, MG1363; 2, MG1363::pCL235; 3, MG1363::pCL237; 4, VEL1122::pCL235; and 5, VEL1122::pCL237. (Electrophoresis conditions: 10 V/cm^−1^/13 h/15 s pulse time in Tris/borate/EDTA 0.05M). As predicted from the physical map of MG1363 chromosome [58] and genome sequence, insertion of integrative plasmids at the *dif*_SL_ site into the 610-kb *Sma*I fragment generated two new *Sma*I fragments of 350- and 260-kb in size. These corresponding restriction fragments were visualized in Southern hybridization using pCL235 and *ilvD* gene as probes in the presence of ^32^P-labeled λ DNA. A part of the chromosome population displayed a WT structure (i.e., with the plasmids pCL235 or pCL237 excised from the chromosome), an instability also observed when transforming E. coli strain with a nonreplicative plasmid containing the *E. coli dif* sequence (F. Cornet, personal communication).
(918 KB PDF)

Figure S2 Phylogenetic Analysis of the 42 Streptococcal Integrases Revealing Two Groups of Conserved Integrases, the XerS Group and the YnbA Group Colors: red, integrases conserved in each streptococcal species; green, phage-related integrases; blue, transposon-related integrases; black, uncharacterized integrases. Bootstrap values are indicated for each branch.
(228 KB PDF)

Table S1 Bacterial Strains and Plasmids (28 KB PDF)

Text S1 Supporting Materials and Methods (16 KB PDF)

### Accession Numbers

The National Center for Biotechnology Information (NCBI) (http://www.ncbi.nlm.nih.gov) accession number for L. lactis is NC_009004.

## Abbreviations

Km^R^: kanamycin resistance
ORF: open reading frame
WT: wild type

## References

[pgen-0030117-b001] Michel B, Grompone G, Flores MJ, Bidnenko V (2004). Multiple pathways process stalled replication forks. Proc Natl Acad Sci U S A.

[pgen-0030117-b002] Steiner W, Kuempel PL (1998). Sister chromatid exchange frequencies in Escherichia coli analyzed by recombination at the *dif* resolvase site. J Bacteriol.

[pgen-0030117-b003] Perals K, Cornet F, Merlet Y, Delon I, Louarn JM (2000). Functional polarization of the Escherichia coli chromosome terminus: The *dif* site acts in chromosome dimer resolution only when located between long stretches of opposite polarity. Mol Microbiol.

[pgen-0030117-b004] Sherratt DJ, Soballe B, Barre FX, Filipe S, Lau I (2004). Recombination and chromosome segregation. Philos Trans R Soc Lond B Biol Sci.

[pgen-0030117-b005] Lesterlin C, Barre FX, Cornet F (2004). Genetic recombination and the cell cycle: What we have learned from chromosome dimers. Mol Microbiol.

[pgen-0030117-b006] Blakely GW, May G, McCulloch R, Arciszewska LK, Burke M (1993). Two related recombinases are required for site-specific recombination at *dif* and *cer* in E. coli K12. Cell.

[pgen-0030117-b007] Hallet B, Arciszewska LK, Sherratt DJ (1999). Reciprocal control of catalysis by the tyrosine recombinases XerC and XerD: An enzymatic switch in site-specific recombination. Mol Cell.

[pgen-0030117-b008] Aussel L, Barre FX, Aroyo M, Stasiak A, Stasiak AZ (2002). FtsK is a DNA motor protein that activates chromosome dimer resolution by switching the catalytic state of the XerC and XerD recombinases. Cell.

[pgen-0030117-b009] Steiner W, Kuempel PL (1998). Cell division is required for resolution of dimer chromosomes at the *dif* locus of Escherichia coli. Mol Microbiol.

[pgen-0030117-b010] Steiner W, Liu G, Donachie WD, Kuempel PL (1999). The cytoplasmic domain of FtsK protein is required for resolution of chromosome dimers. Mol Microbiol.

[pgen-0030117-b011] Recchia GD, Aroyo M, Wolf D, Blakely GW, Sherratt DJ (1999). FtsK-dependent and -independent pathways of Xer site-specific recombination. EMBO J.

[pgen-0030117-b012] Barre FX, Aroyo M, Colloms SD, Helfrich A, Cornet F (2000). FtsK functions in the processing of a Holliday junction intermediate during bacterial chromosome segregation. Genes Dev.

[pgen-0030117-b013] Wang L, Lutkenhaus J (1998). FtsK is an essential cell division protein that is localized to the septum and induced as part of the SOS response. Mol Microbiol.

[pgen-0030117-b014] Corre J, Louarn JM (2002). Evidence from terminal recombination gradients that FtsK uses replichore polarity to control chromosome terminus positioning at division in Escherichia coli. J Bacteriol.

[pgen-0030117-b015] Capiaux H, Lesterlin C, Perals K, Louarn JM, Cornet F (2002). A dual role for the FtsK protein in Escherichia coli chromosome segregation. EMBO Rep.

[pgen-0030117-b016] Ip SC, Bregu M, Barre FX, Sherratt DJ (2003). Decatenation of DNA circles by FtsK-dependent Xer site-specific recombination. EMBO J.

[pgen-0030117-b017] Levy O, Ptacin JL, Pease PJ, Gore J, Eisen MB (2005). Identification of oligonucleotide sequences that direct the movement of the Escherichia coli FtsK translocase. Proc Natl Acad Sci U S A.

[pgen-0030117-b018] Bigot S, Saleh OA, Lesterlin C, Pages C, El Karoui M (2005). KOPS: DNA motifs that control E. coli chromosome segregation by orienting the FtsK translocase. EMBO J.

[pgen-0030117-b019] Bigot S, Saleh OA, Cornet F, Allemand JF, Barre FX (2006). Oriented loading of FtsK on KOPS. Nat Struct Mol Biol.

[pgen-0030117-b020] Yates J, Zhekov I, Baker R, Eklund B, Sherratt DJ (2006). Dissection of a functional interaction between the DNA translocase, FtsK, and the XerD recombinase. Mol Microbiol.

[pgen-0030117-b021] Yates J, Aroyo M, Sherratt DJ, Barre FX (2003). Species specificity in the activation of Xer recombination at *dif* by FtsK. Mol Microbiol.

[pgen-0030117-b022] Recchia GD, Sherratt DJ (1999). Conservation of *xer* site-specific recombination genes in bacteria. Mol Microbiol.

[pgen-0030117-b023] Hendrickson H, Lawrence JG (2007). Mutational bias suggests that replication termination occurs near the *dif* site, not at Ter sites. Mol Microbiol.

[pgen-0030117-b024] Neilson L, Blakely GW, Sherratt DJ (1999). Site-specific recombination at *dif* by Haemophilus influenzae XerC. Mol Microbiol.

[pgen-0030117-b025] Blakely GW, Davidson AO, Sherratt DJ (2000). Sequential strand exchange by XerC and XerD during site-specific recombination at *dif*. J Biol Chem.

[pgen-0030117-b026] Sciochetti SA, Piggot PJ, Blakely GW (2001). Identification and characterization of the *dif* Site from Bacillus subtilis. J Bacteriol.

[pgen-0030117-b027] Villion M, Szatmari G (2003). The XerC recombinase of Proteus mirabilis: Characterization and interaction with other tyrosine recombinases. FEMS Microbiol Lett.

[pgen-0030117-b028] Jouan L, Szatmari G (2003). Interactions of the Caulobacter crescentus XerC and XerD recombinases with the *E. coli dif* site. FEMS Microbiol Lett.

[pgen-0030117-b029] Mitchell TJ (2003). The pathogenesis of streptococcal infections: From tooth decay to meningitis. Nat Rev Microbiol.

[pgen-0030117-b030] Hols P, Hancy F, Fontaine L, Grossiord B, Prozzi D (2005). New insights in the molecular biology and physiology of Streptococcus thermophilus revealed by comparative genomics. FEMS Microbiol Rev.

[pgen-0030117-b031] Hugenholtz J, Smid EJ (2002). Nutraceutical production with food-grade microorganisms. Curr Opin Biotechnol.

[pgen-0030117-b032] Chalker AF, Lupas A, Ingraham K, So CY, Lunsford RD (2000). Genetic characterization of gram-positive homologs of the XerCD site-specific recombinases. J Mol Microbiol Biotechnol.

[pgen-0030117-b033] Reichmann P, Hakenbeck R (2002). A XerD recombinase with unusual active site motifs in Streptococcus pneumoniae. J Mol Microbiol Biotechnol.

[pgen-0030117-b034] Grigoriev A (1998). Analyzing genomes with cumulative skew diagrams. Nucleic Acids Res.

[pgen-0030117-b035] Rocha EP (2004). The replication-related organization of bacterial genomes. Microbiology.

[pgen-0030117-b036] Bolotin A, Wincker P, Mauger S, Jaillon O, Malarme K (2001). The complete genome sequence of the lactic acid bacterium Lactococcus lactis ssp. *lactis* IL1403. Genome Res.

[pgen-0030117-b037] Perals K, Capiaux H, Vincourt JB, Louarn JM, Sherratt DJ (2001). Interplay between recombination, cell division and chromosome structure during chromosome dimer resolution in Escherichia coli. Mol Microbiol.

[pgen-0030117-b038] Ferreira H, Butler-Cole B, Burgin A, Baker R, Sherratt DJ (2003). Functional analysis of the C-terminal domains of the site-specific recombinases XerC and XerD. J Mol Biol.

[pgen-0030117-b039] Leslie NR, Sherratt DJ (1995). Site-specific recombination in the replication terminus region of *Escherichia coli:* Functional replacement of *dif*. EMBO J.

[pgen-0030117-b040] Iyer LM, Makarova KS, Koonin EV, Aravind L (2004). Comparative genomics of the FtsK-HerA superfamily of pumping ATPases: Implications for the origins of chromosome segregation, cell division and viral capsid packaging. Nucleic Acids Res.

[pgen-0030117-b041] Mazel D (2006). Integrons: Agents of bacterial evolution. Nat Rev Microbiol.

[pgen-0030117-b042] Sternberg N, Sauer B, Hoess R, Abremski K (1986). Bacteriophage P1 cre gene and its regulatory region. Evidence for multiple promoters and for regulation by DNA methylation. J Mol Biol.

[pgen-0030117-b043] Lee MH, Pascopella L, Jacobs WR, Hatfull GF (1991). Site-specific integration of mycobacteriophage L5: Integration-proficient vectors for Mycobacterium smegmatis, Mycobacterium tuberculosis, and bacille Calmette-Guerin. Proc Natl Acad Sci U S A.

[pgen-0030117-b044] Ravatn R, Studer S, Zehnder AJ, van der Meer JR (1998). Int-B13, an unusual site-specific recombinase of the bacteriophage P4 integrase family, is responsible for chromosomal insertion of the 105-kilobase clc element of Pseudomonas sp. Strain B13. J Bacteriol.

[pgen-0030117-b045] Cheng Q, Paszkiet BJ, Shoemaker NB, Gardner JF, Salyers AA (2000). Integration and excision of a *Bacteroides* conjugative transposon, CTnDOT. J Bacteriol.

[pgen-0030117-b046] Makarova KS, Koonin EV (2007). Evolutionary genomics of lactic acid bacteria. J Bacteriol.

[pgen-0030117-b047] Henz SR, Huson DH, Auch AF, Nieselt-Struwe K, Schuster SC (2005). Whole-genome prokaryotic phylogeny. Bioinformatics.

[pgen-0030117-b048] Huber KE, Waldor MK (2002). Filamentous phage integration requires the host recombinases XerC and XerD. Nature.

[pgen-0030117-b049] Dillard JP, Seifert HS (2001). A variable genetic island specific for Neisseria gonorrhoeae is involved in providing DNA for natural transformation and is found more often in disseminated infection isolates. Mol Microbiol.

[pgen-0030117-b050] Van Duyne GD (2001). A structural view of Cre-*loxP* site-specific recombination. Annu Rev Biophys Biomol Struct.

[pgen-0030117-b051] Chen Y, Rice PA (2003). New insight into site-specific recombination from Flp recombinase-DNA structures. Annu Rev Biophys Biomol Struct.

[pgen-0030117-b052] Wu LJ, Errington J (1997). Septal localization of the SpoIIIE chromosome partitioning protein in Bacillus subtilis. EMBO J.

[pgen-0030117-b053] Begg KJ, Dewar SJ, Donachie WD (1995). A new Escherichia coli cell division gene, *ftsK*. J Bacteriol.

[pgen-0030117-b054] Wang SC, West L, Shapiro L (2006). The bifunctional FtsK protein mediates chromosome partitioning and cell division in *Caulobacter*. J Bacteriol.

[pgen-0030117-b055] Bigot S, Corre J, Louarn JM, Cornet F, Barre FX (2004). FtsK activities in Xer recombination, DNA mobilization and cell division involve overlapping and separate domains of the protein. Mol Microbiol.

[pgen-0030117-b056] Le Bourgeois P, Langella P, Ritzenthaler P, Teissié J, Eynard N (2000). Electrotransformation of Lactococcus lactis. Electrotransformation of bacteria, Springer Lab Manual.

[pgen-0030117-b057] Alloing G, Granadel C, Morrison DA, Claverys JP (1996). Competence pheromone, oligopeptide permease, and induction of competence in Streptococcus pneumoniae. Mol Microbiol.

[pgen-0030117-b058] Le Bourgeois P, Lautier M, van den Berghe L, Gasson MJ, Ritzenthaler P (1995). Physical and genetic map of the Lactococcus lactis subsp. *cremoris* MG1363 chromosome: Comparison with that of Lactococcus lactis subsp. *lactis* IL1403 reveals a large genome inversion. J Bacteriol.

[pgen-0030117-b059] Schwartz S, Elnitski L, Li M, Weirauch M, Riemer C (2003). MultiPipMaker and supporting tools: Alignments and analysis of multiple genomic DNA sequences. Nucleic Acids Res.

[pgen-0030117-b060] Maguin E, Prévots H, Ehrlich SD, Gruss A (1996). Efficient insertional mutagenesis in *Lactococci* and other Gram-positive bacteria. J Bacteriol.

[pgen-0030117-b061] Prudhomme M, Camilli A, Claverys JP, Hakenbeck R, Chhatwal GS (2006). In vitro *mariner* mutagenesis of Streptococcus pneumoniae: Tools and traps. The molecular biology of Streptococci.

[pgen-0030117-b062] Gutierrez J, Larsen R, Cintas LM, Kok J, Hernandez PE (2006). High-level heterologous production and functional expression of the sec-dependent enterocin P from Enterococcus faecium P13 in Lactococcus lactis. Appl Microbiol Biotechnol.

[pgen-0030117-b063] Lewis PJ, Marston AL (1999). GFP vectors for controlled expression and dual labelling of protein fusions in Bacillus subtilis. Gene.

[pgen-0030117-b064] Kuipers OP, Beerthuyzen MM, Siezen RJ, de Vos WM (1993). Characterization of the nisin gene cluster *nisABTCIPR* of Lactococcus lactis. Requirement of expression of the *nisA* and *nisI* genes for development of immunity. Eur J Biochem.

