# Supplementary material for: The Unconventional Xer Recombination Machinery of Streptococci/Lactococci
Source: PLoS Genet. 2007 Jul 13;3(7):e117. doi: 10.1371/journal.pgen.0030117 (PMC1914069; doi:10.1371/journal.pgen.0030117)
Supplement: Supplementary file 1 [file pgen.0030117.sd001.pdf]

Supporting Materials and Methods

pGh9-derivative plasmids and *E. coli* strains constructions

pGh9-derivative plasmids were constructed as follows: relevant pairs of oligonucleotides containing *Xma*I linkers were annealed together, generating DNA duplexes with two *Xma*I-compatible ends. Only the left end of the duplexes recreated a full *Xma*I site, allowing further determination of its orientation by restriction analysis (only the synthetic sequences cloned in counterclockwise orientation were selected for experiments). These duplexes were 5’-phosphorylated and ligated with dephosphorylated *Xma*I-digested pCL52 or pGh9 vectors. The sequence of each cloned oligonucleotide was confirmed by DNA sequencing. The *dif*-Km^{R}-*dif* cassette was introduced in *E. coli* strain LN2772 by integration-excision experiment [1], using pCL294 plasmid (Table S1). *E. coli* E359-derivative mutants carrying *xerC2*::Ap, *xerD*::Ap, or *ftsK*$_{C}$::Ap were obtained by P1-mediated transduction using phage lysates kindly provided by F. Cornet. The *ftsK*$_{C}$::Ap disruption corresponds to the replacement of *Fsp*I internal fragments of *ftsK* by the *bla* gene, and allows the synthesis of only the 316 first amino acids of FtsK (F. Cornet, personal communication). Allelic replacements were verified by PCR, and the *xer*$^{-}$ phenotype was tested by *in vivo* plasmid resolution assay using plasmid pFX142 [2]. The *recA56* allele was introduced by conjugation by co-transfer with *srl*::Tn*10* from strain JC10240 [3], and the *recA* phenotype was verified by UV-radiation sensitivity.

Phylogenetic analysis of the streptococcal tyrosine recombinases

Tyrosine recombinase protein sequences were obtained from BLASTp searches (http://www.ncbi.nlm.nih.gov/sutils/genom_tree.cgi?) against ten streptococcal genomes (strains; *S. agalactiae* NEM316 and 2603V/R, *S. pneumoniae* TIGR4 and R6, *S. mutans*

UA159, *S. pyogenes* M1 GAS, M315, M8232, and SSI-1, and *L. lactis* IL1403), using *E. faecalis* CodV (XerC-like), *E. faecalis* RipX (XerD-like), and *S. pneumoniae* XerD (spr1692) sequences as queries. A total of 65 putative integrases were identified, and proteins from the same streptococcal species with >99% of amino acids identities were considered as duplicate and removed from the analysis. The remaining 42 non-redundant streptococcal integrases and the *E. faecalis* CodV and RipX sequences were aligned using CLUSTALX ver. 1.81 [4]. Phylogenic trees were constructed using PHYLIP 3.6 package [5]. The CLUSTALX alignment was run with the PROTDIST program with default settings to identify separation distances, and the calculated distances used as input file for NEIGHBOR program, with the *E. faecalis* CodV forced as outgroup. Bootstrap values were determined by performing SEQBOOT (500 repetitions) of the CLUSTALX alignment. The resulting file was used as input for PROTDIST program, with a 44 times randomization of the input order of sequences. A consensus tree was determined from the 500 best trees generated, and branches with bootstrap values <50% were collapsed together.

References for Supporting Materials and Methods

1. Corre J, Cornet F, Patte J, Louarn JM (1997) Unraveling a region-specific hyper-recombination phenomenon: genetic control and modalities of terminal recombination in *Escherichia coli*. Genetics 147: 979-989.

2. Aussel L, Barre FX, Aroyo M, Stasiak A, Stasiak AZ et al. (2002) FtsK is a DNA motor protein that activates chromosome dimer resolution by switching the catalytic state of the XerC and XerD recombinases. Cell 108: 195-205.

3. Capiaux H, Lesterlin C, Perals K, Louarn JM, Cornet F (2002) A dual role for the FtsK protein in *Escherichia coli* chromosome segregation. EMBO Rep 3: 532-536.

4. Thompson JD, Gibson TJ, Plewniak F, Jeanmougin F, Higgins DG (1997) The CLUSTAL_X windows interface: flexible strategies for multiple sequence alignment aided by quality analysis tools. Nucleic Acids Res 25: 4876-4882.

5. Felsenstein J (1989) PHYLIP - Phylogeny Inference Package (Version 3.2). Cladistics 5: 164-166.
