# Supplementary material for: The Unconventional Xer Recombination Machinery of Streptococci/Lactococci
Source: PLoS Genet. 2007 Jul 13;3(7):e117. doi: 10.1371/journal.pgen.0030117 (PMC1914069; doi:10.1371/journal.pgen.0030117)
Supplement: Supplementary file 2 [file pgen.0030117.sg001.pdf]

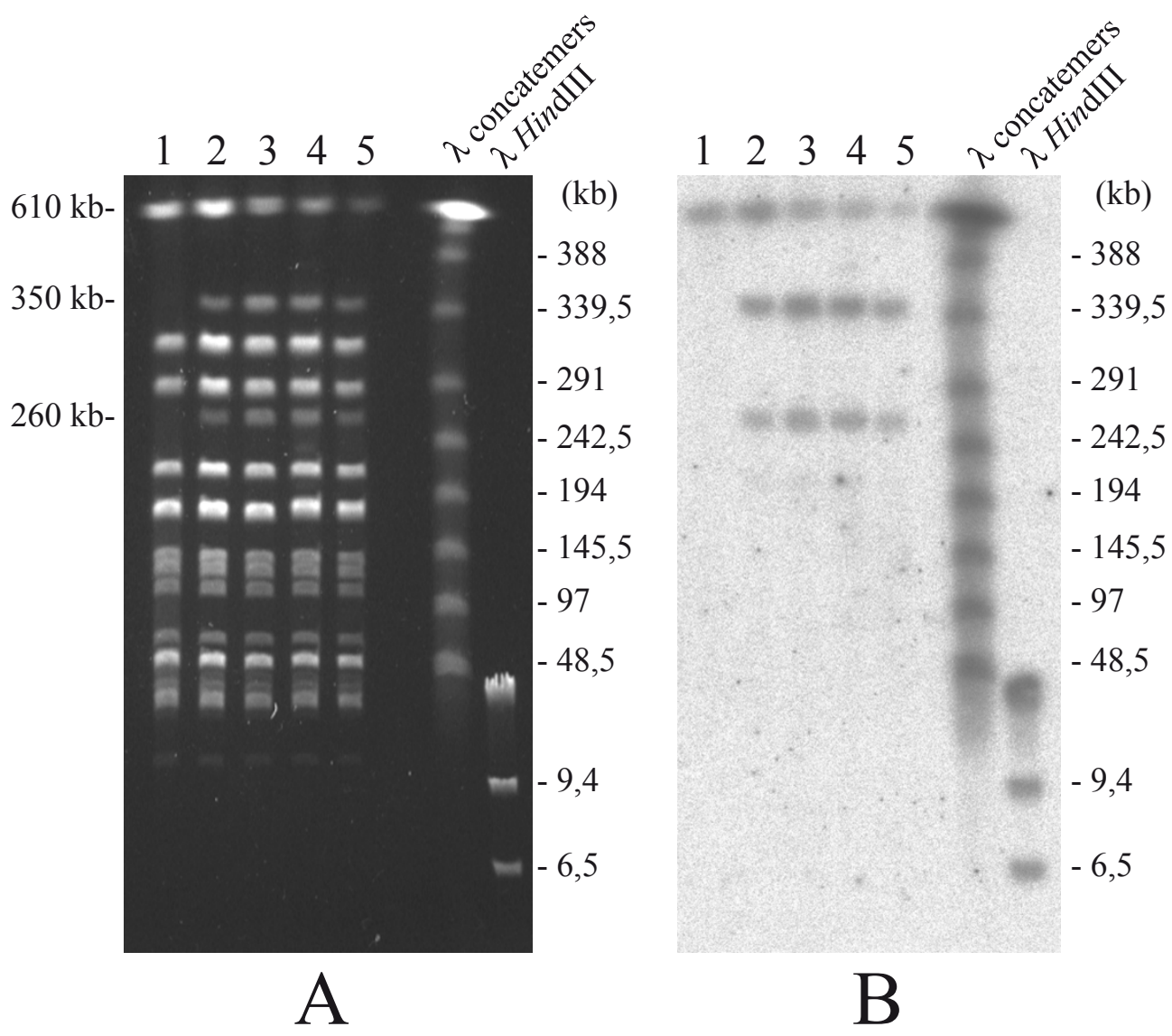
1 2 3 4 5
λ concatemers
λ HindIII
(kb)
610 kb-
350 kb-
260 kb-
- 388
- 339,5
- 291
- 242,5
- 194
- 145,5
- 97
- 48,5
- 9,4
- 6,5
A
B

Fig. S1
