# Supplementary material for: The Unconventional Xer Recombination Machinery of Streptococci/Lactococci
Source: PLoS Genet. 2007 Jul 13;3(7):e117. doi: 10.1371/journal.pgen.0030117 (PMC1914069; doi:10.1371/journal.pgen.0030117)
Supplement: Supplementary file 3 [file pgen.0030117.sg002.pdf]

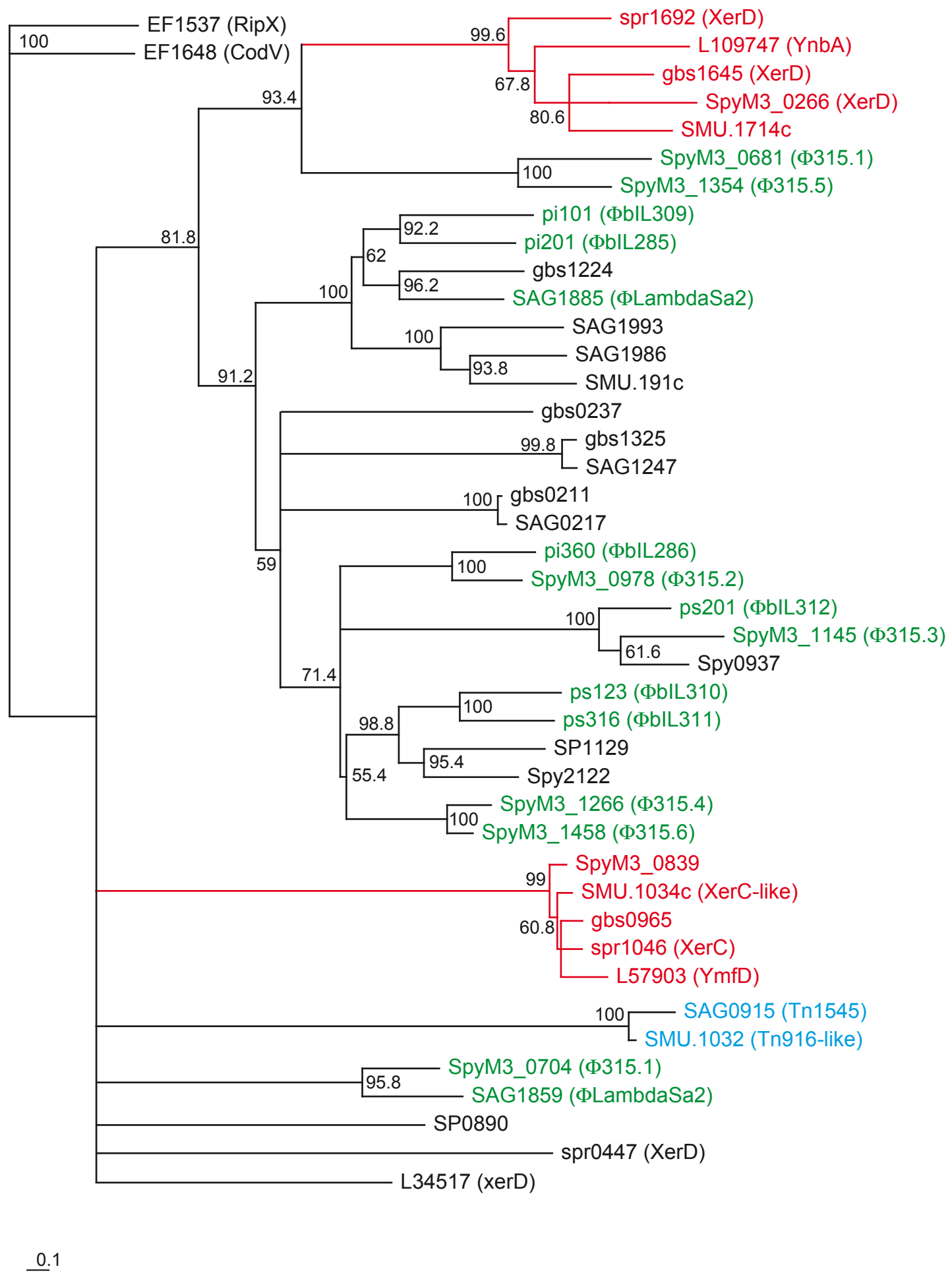
EF1537 (RipX)
EF1648 (CodV)
100
spr1692 (XerD)
L109747 (YnbA)
gbs1645 (XerD)
SpyM3_0266 (XerD)
SMU.1714c
99.6
67.8
80.6
93.4
SpyM3_0681 (Φ315.1)
SpyM3_1354 (Φ315.5)
100
81.8
pi101 (ΦbIL309)
pi201 (ΦbIL285)
92.2
62
gbs1224
SAG1885 (ΦLambdaSa2)
96.2
100
SAG1993
SAG1986
SMU.191c
100
93.8
91.2
gbs0237
gbs1325
SAG1247
99.8
gbs0211
SAG0217
100
59
pi360 (ΦbIL286)
SpyM3_0978 (Φ315.2)
100
ps201 (ΦbIL312)
SpyM3_1145 (Φ315.3)
Spy0937
100
61.6
71.4
ps123 (ΦbIL310)
ps316 (ΦbIL311)
100
98.8
SP1129
Spy2122
95.4
55.4
SpyM3_1266 (Φ315.4)
SpyM3_1458 (Φ315.6)
100
SpyM3_0839
SMU.1034c (XerC-like)
99
gbs0965
spr1046 (XerC)
L57903 (YmfD)
60.8
SAG0915 (Tn1545)
SMU.1032 (Tn916-like)
100
SpyM3_0704 (Φ315.1)
SAG1859 (ΦLambdaSa2)
95.8
SP0890
spr0447 (XerD)
L34517 (xerD)
0.1

Fig. S2
