# Supplementary material for: The Unconventional Xer Recombination Machinery of Streptococci/Lactococci
Source: PLoS Genet. 2007 Jul 13;3(7):e117. doi: 10.1371/journal.pgen.0030117 (PMC1914069; doi:10.1371/journal.pgen.0030117)
Supplement: Supplementary file 4 [file pgen.0030117.st001.pdf]

TABLE S1. Bacterial strains and plasmids

Plasmid or strain	Relevant properties	Origin
**Plasmids**		
pCL52	Em^R, a 2.4-kb *E. coli* cloning vector derived from pBluescript KSII	[1]
PGh9	Em^R, a 3.7-kb lactococcal *repA* ^{ts} cloning vector	[2]
pFC23	Cm^R, pKO3-derivative plasmid (*repA*^{ts}) containing the *rpsL*$^+$ gene. Used for replacement of the *E. coli dif* site by allelic exchange	[3]
pFX170	Ap^R, full-length *E. coli* FtsK protein fused to a FLAG epitope on its N-terminus and cloned into pBAD24 vector	[4]
pFX144	Cm^R, pKO3-derivative (*repA*^{ts}) plasmid. Used for allelic exchange at *xerC* locus in *E. coli*	[5]
pCL231	Em^R, pGh9-derivative (*repA*^{ts}) plasmid. Contains a 37-bp fragment, *dif*-2 (5'-TAAC**ATCTTTCCgAAAAACTgTAA TTTTCTTgACA**AT-3') cloned at the *Xma*I site (the 31-bp lactococcal *dif*$_{SL}$ site is indicated in bold)	This work
pCL235	Em^R, pCL52-derivative plasmid. Contains the same 37-bp synthetic fragment than pCL231	This work
pCL237	Em^R, pCL52-derivative plasmid. Contains a 27-bp lactococcal *dif*$_{SL}$ variant, *dif*-8 (5'-**CTTTCCgAAAAACT gTAATTTTCTTgA**-3') of pCL233 (see Table 2) cloned at the *Xma*I site	This work
pCL263	Em^R, 5.35-kb *Xba*I-*Cla*I fragment from pFX170 (containing	This work

	the FLAG-tagged full-length *ftsK* gene of *E. coli*, the araBAD promoter, and the *araC* gene) cloned at the corresponding sites of pCL52	
pCL294	Cm^{R}, 2.04-kb *dif*-2-Km^{R}-*dif*-2 cassette cloned at the *Xmn*I site of pFC23. Used for introducing the cassette at the *E. coli dif* locus	This work
pCL297	Cm^{R}, lactococcal *xerS* ORF (*ymfD*) cloned into one *Nco*I site of pFX144. *xerS* is expressed from transcriptional read-through of the upstream *cat* gene	This work
pCL403	Em^{R}, pGh9-derivative (*repA*^{ts}) plasmid. Contains a 37-bp fragment (5'-TAAC**ATCTTTCCgAAAAACTATAATTT TCTTgAAA**AA-3') cloned at the *Xma*I site (the 31-bp pneumococcal *dif*$_{SL}$ site is indicated in bold)	This work
pKNtergfp	Em^{R}, 181 first amino acids of the lactococcal FtsK protein fused to Gfpmut1 and expressed from P$_{nisA}$ of pNG8048e vector	N. Campo, laboratory collection
pKFLgfp	Em^{R}, full-length lactococcal FtsK protein fused to Gfpmut1 and expressed from P$_{nisA}$ of pNG8048e vector	N. Campo, laboratory collection
**Strains**		
L. lactis		
MG1363	Wild Type strain. Plasmid-free derivative strain of NCDO712	[6]
VEL1122	Tc^{R}, MG1363 *recA*::*tetM*	[7]
NZ9000	MG1363 *pepN*::*nisRK*. Recipient strain for the nisin expression system.	[8]

S. pneumoniae		
R800	Wild Type strain. Derivative of strain R6	[9]
S501	R800 *xerS* (spr1046::*Km*)	This work
S502	R800 $ftsK_C$ (spr0781::*Km*)	This work
E. coli		
LN2772	Str^R, Tc^R, W1485 *leu thyA deoB* or *C supE rpsL* Δ(*dif*)58::Tc	[3]
LN3038	Str^R, Tc^R, W1485 *leu thyA deoB* or *C supE rpsL lacZ*::*Tn*10. This strain has a wild type phenotype for XerCD/*dif* recombination.	F. Cornet, laboratory collection
E359	Str^R, Km^R, LN2772 with the lactococcal *dif*-2-Km^R-*dif*-2 cassette inserted in the *tetA* gene	This work
E367	Str^R, Tc^R, Cm^R, LN2772 containing pCL297	This work
E368	Str^R, Cm^R, E359 with one dif_{SL} site (the Km^R cassette has been excised by XerS/dif_{SL} recombination) and containing pCL297	This work
E372	Str^R, Km^R, Ap^R, E359 $ftsK_C$::Ap	This work
E375	Str^R, Tc^R, Cm^R, LN3038 containing pCL297	This work
E378	Str^R, Ap^R, Cm^R, E372 with one dif_{SL} site (the Km^R cassette has been excised by XerS/dif_{SL} recombination) and containing pCL297	This work
E379	Str^R, Km^R, Tc^R, E359 *recA*56	This work
E408	Str^R, Km^R, Ap^R, Tc^R, E359 *xerC*::Ap	This work
E409	Str^R, Km^R, Ap^R, Tc^R, E359 *xerD*::Ap	This work

References for Table S1

1. Campo N, Daveran-Mingot M-L, Leenhouts KJ, Ritzenthaler P, Le Bourgeois P (2002) Cre/*loxP* recombination system for large genome rearrangements in *Lactococcus lactis*. Appl Environ Microbiol 68: 2359-2367.

2. Maguin E, Prévots H, Ehrlich SD, Gruss A (1996) Efficient insertional mutagenesis in *Lactococci* and other Gram-positive bacteria. J Bacteriol 178: 931-935.

3. Cornet F, Mortier I, Patte J, Louarn JM (1994) Plasmid pSC101 harbors a recombination site, *psi*, which is able to resolve plasmid multimers and to substitute for the analogous chromosomal *Escherichia coli* site *dif*. J Bacteriol 176: 3188-3195.

4. Yates J, Aroyo M, Sherratt DJ, Barre FX (2003) Species specificity in the activation of Xer recombination at *dif* by FtsK. Mol Microbiol 49: 241-249.

5. Aussel L, Barre FX, Aroyo M, Stasiak A, Stasiak AZ et al. (2002) FtsK is a DNA motor protein that activates chromosome dimer resolution by switching the catalytic state of the XerC and XerD recombinases. Cell 108: 195-205.

6. Gasson MJ (1983) Plasmid complements of *Streptococcus lactis* NCDO712 and other lactic *Streptococci* after protoplast-induced curing. J Bacteriol 154: 1-9.

7. Duwat P, Ehrlich SD, Gruss A (1995) The *recA* gene in *Lactococcus lactis*: characterization and involvement in oxydative and thermal stress. Mol Microbiol 17: 1121-1131.

8. Kuipers OP, de Ruyter PG, Kleerebezem M, de Vos WM (1998) Quorum sensing-controlled gene expression in lactic acid bacteria. J Biotechnol 64: 15-21.

9. Lefevre JC, Claverys JP, Sicard AM (1979) Donor deoxyribonucleic acid length and marker effect in pneumococcal transformation. J Bacteriol 138: 80-86.
